# Cross-cultural adaptation and content validation of START: Adaptação transcultural e validação de conteúdo do START

**DOI:** 10.1590/1516-3180.2014.00303101

**Published:** 2016-01-19

**Authors:** Aline Cristina Luz, Márcio Galvão Oliveira, Lúcia Noblat

**Affiliations:** I BPharm. Clinical Pharmacist, Cardiopulmonary Institute, Salvador, Bahia, Brazil.; II BPharm, PhD. Adjunct Professor of Clinical Pharmacy, Multidisciplinary Health Institute, Universidade Federal da Bahia (UFBA), Vitória da Conquista, Bahia, Brazil.; III BPharm, PhD. Associate Professor of Clinical Pharmacy, School of Pharmacy, Universidade Federal da Bahia (UFBA), Salvador, Bahia, Brazil.

**Keywords:** Elderly, Pharmaceutical preparations, Drug prescriptions, Health of the elderly, Drug utilization, Translations

## Abstract

**CONTEXT AND OBJECTIVE:**

Non-treatment of diseases or clinical conditions has been considered to constitute omission of care in several countries. The aim of the present study was to develop a transcultural adaptation of the Screening Tool to Alert Doctors to the Right Treatment (START) to Brazilian Portuguese and to validate the tool’s content.

**DESIGN AND SETTING:**

Cultural adaptation and validation of the START criteria using the Delphi consensus technique.

**METHOD:**

START was translated from its original language into Brazilian Portuguese, followed by back-translation and validation by means of the modified Delphi technique. For this, an electronic form was developed and sent to 20 experts, who were asked to use a Likert scale to assess the statements included in START, in relation to their pertinence to Brazilian realities. All of the statements that exhibited mean scores greater than 4.0 were considered to have attained consensus. The experts’ identities were kept confidential throughout the validation process.

**RESULTS:**

In the first phase of the validation process, 63.6% (14/22) of the statements in START attained consensus. The remaining statements were returned to the experts so that they could have the opportunity to review their comments and statements and to assess them again, based on the Likert scale used earlier. In this phase, 100% of the START instrument attained consensus.

**CONCLUSION:**

The content of START was entirely validated for Brazil, with all of the original criteria maintained.

## RESUMO

**CONTEXTO E OBJETIVO:**

O não tratamento de doenças ou condições clínicas tem sido apontado como omissão no cuidado em diversos países. O objetivo deste estudo foi realizar a adaptação transcultural para o português brasileiro e sua validação de conteúdo do questionário START (Screening Tool to Alert Doctors to the Right Treatment).

**TIPO DE ESTUDO E LOCAL:**

Adaptação cultural e validação dos critérios START através da técnica de consenso Delphi.

**MÉTODO:**

O START foi submetido à tradução da língua original para o Português (Brasil), tradução reversa e validação de conteúdo por meio da técnica Delphi modificada. Para isso, um formulário eletrônico foi desenvolvido e enviado a 20 especialistas para o julgamento das proposições presentes na ferramenta START conforme sua pertinência para a realidade brasileira, utilizando uma escala de Likert. Foram consideradas consensuais todas as proposições que apresentaram média superior a 4,0. A identificação de cada especialista foi mantida em confidencialidade durante o todo o processo.

**RESULTADOS:**

Na primeira fase da validação, foi obtido consenso em 63,6% (14/22) das proposições contidas na ferramenta START. As proposições não consensuais foram reencaminhadas para os especialistas, que tiveram a oportunidade de rever seus comentários e proposições e julgá-las com base na escala de Likert utilizada previamente. Nessa fase, foi obtido consenso em 100% do instrumento START.

**CONCLUSÃO:**

O conteúdo do START foi validado para o Brasil na sua totalidade, com todos os critérios originais mantidos.

## INTRODUCTION

Older adults are a heterogeneous group that usually presents a large number of chronic diseases, thus leading these individuals to use healthcare services and medications frequently.^1^ Therefore, several researchers have formulated instruments to assess the appropriateness of drug prescribing among older adults.^2^ According to Barry et al.,^3^ a drug is rated inappropriate for older people when their tolerance to it has been scientifically and clinically shown to be poor, due to the physiological changes associated with aging. Such drugs may even exacerbate clinical problems. Page and Ruscin^4^ considered a prescription to be inappropriate when it exhibited a significant risk of causing adverse events or when there was evidence that equally or more effective and safer alternatives existed for treating the same condition.

Most instruments published to date within this field have assessed inappropriate prescribing of medication to older adults, but few have evaluated errors of omission with regard to prescriptions.^3^ START (Screening Tool to Alert Doctors to the Right Treatment) was formulated with the aim of detecting prescribing omissions among elderly patients. This instrument provides a method for systematic detection of prescribing omissions based on physiological systems, and it is considered to be valid, effective and easy to use.^5^ The START criteria were formulated and validated in 2006 in the United Kingdom using the Delphi^6^ consensus technique. The START criteria include 22 indicators of potential prescribing omissions among older adults, and its use for both outpatients and inpatients has become widespread across Europe.^7^

## OBJECTIVE

The aim of the present study was to develop and validate a cross-cultural Brazilian Portuguese adaptation of START.

## METHODS

### Instrument

START is a published, evidence-based screening tool for detecting potential prescribing omissions among elderly patients. START categorizes prescribing omissions according to physiological systems within the following fields: cardiology, endocrinology, rheumatology, pneumology and neurology. The tool includes 22 indicators of potential prescribing omissions among older adults, but does not provide scores for each indicator.

### Translation and cross-cultural adaptation

START was published by Gallagher et al.^5^ Although this tool is in the public domain, the main author was contacted by e-mail to seek permission to adapt the content to Brazilian Portuguese and validate the adapted tool, and this permission was granted.

Firstly, the original version of the START criteria (in English) was translated into Brazilian Portuguese by a sworn translator who is a native Portuguese speaker. This translation was called version 1. This was then translated into English by a second translator, to produce a back-translation called version 2. This method of translation and transcultural adaptation followed the methodology of Guillemin et al.^8^ Both of these translators were blinded to the study aims. The Delphi method^6^ was used to validate the instrument.

The translations were compared by the authors of the present article. The three authors evaluated them independently. Inconsistencies were resolved by consensus to produce a START version in the Brazilian Portuguese language.

### Validation of the instrument’s content

The validation study was conducted in 2013, and it included Brazilian experts in the areas of geriatrics, cardiology, endocrinology, neurology, pulmonology and rheumatology. The modified Delphi technique was used to validate the instrument’s content.^6^

The participating experts were specialists in their fields. They were living and working in Brazil and were known for their medical expertise and scientific production. Each participant was sent an invitation letter by e-mail that explained the study aims and the consensus technique that would be used.

After the participants signed an informed consent form, each of them was sent the electronic version of START by e-mail (Delphi round 1), taking their clinical specialization into consideration. Thus, before actually filling out the electronic form, each participant was ask to indicate his or her field of specialization, which granted him or her access to the statements relevant to his or her specialty only. Only the geriatrists had access to the full content of the instrument, which consisted of 22 statements.

The participants were asked to judge the information relating to the clinical situations described in the questionnaire and to record the answer that they considered to be most pertinent: 1. I fully disagree; 2. I partially disagree; 3. Indifferent; 4. I partially agree; or 5. I fully agree. Responses to each statement in START regarding prescribing omissions were provided by eight experts, namely five geriatrists and three specialists in the corresponding field.

Following the counting of responses and processing of comments, statements scoring less than the preset cutoff point (mean: 4.0), exhibiting confidence intervals (95% CI) less than the cutoff point and/or receiving substantial comments, as well as those for which changes in the information provided were requested, were sent back for reassessment (Delphi round 2).

Each participant was then given the opportunity to revise or confirm his or her previous position relative to the statements sent back for revision. During the first two *Delphi* rounds, the participants were not informed of the identities of the remainder of the participants. A statement was considered to have attained consensus in round 2 when its mean score and 95% CI were higher than 4.0 (i.e. the cutoff point representing 80% agreement). When a statement score was less than 4.0 in round 2, because of the addition of comments made in round 1, the original content was maintained.

The data analysis included calculation of the mean scores assigned to each statement and the corresponding 95% CI. Analysis was performed using the SPSS software, version 22.0 (Chicago, IL, USA).

The study was approved by a local ethics committee.

## RESULTS

Twenty-two experts were invited to participate in the study, and 20 agreed to participate, namely five geriatrists, three cardiologists, three endocrinologists, three pulmonologists, three neurologists and three rheumatologists. Approximately 60% (12/20) of the participants had a doctoral degree, 30% (6/20) had a master’s degree and 10% (2/20) were accredited specialists only.

The first phase of the validation process lasted two months. No consensus was reached for eight of the 22 statements (36.4%). One of the geriatrists dropped out of the study for personal reasons during round two. The second phase also lasted two months, and consensus was reached for all eight statements discussed. The results relating to both Delphi rounds are presented in Table 1.

The START version validated and adapted for Brazilian realities is described in the Supplementary Material (in English and Portuguese).

**Table 1.**
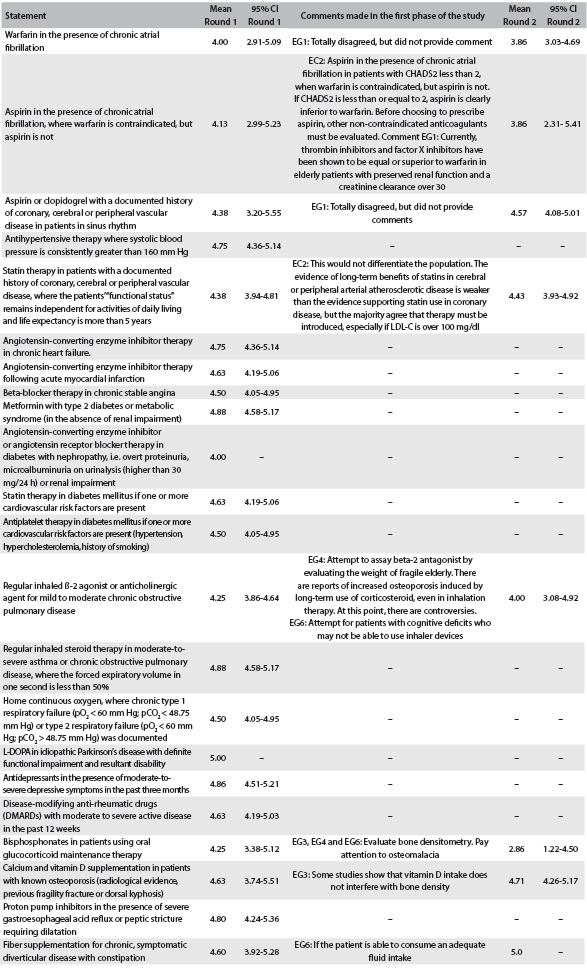
Scores from the first and second phases of START content validation, by means of the modified Delphi technique

Legend: CI = confidence interval; renal dysfunction = glomerular filtration rate < 50 ml/minute; EC2 = Expert Cardiologist 2; EG1 = Expert Geriatrist 1; EG3 =: Expert Geriatrist 3; EG4 = Expert Geriatrist 4; EG6 = Expert Geriatrist 6; LDL = low-density lipoprotein cholesterol

## DISCUSSION

No consensus was reached for 36.4% of the START statements in the first round of the Delphi survey. In the second round, all of the statements reached consensus. The likely reason for this finding was that although the START statements were published eight years ago, they exhibit current high-quality evidence supporting use of the drugs mentioned among older adults.

Several techniques are available for validating the content of clinical criteria, instruments or questionnaires. The Delphi technique aims to refine the opinions of an expert panel to achieve a consensus, by means of several questionnaires with controlled feedback.^6^ It is a systematic method based on the participants’ clinical experience, and therefore the level of expertise among the participants needs to be assessed before its application.^3^^,^^6^^,^^9^ All of the START fields of expertise were assessed by a minimum of eight experts. The participants selected were well known for their medical expertise, as well as for the number of scientific articles that they had published in Brazilian and international journals. In addition, all of the participants had had significant experience in relation to care for older adults, and they were affiliated to higher education institutions. These features make it possible to obtain highly qualified contributions and to gather together experts with different experiences encompassing several specialties.

Two sworn translators were selected to perform the forward translation (first translator to the Brazilian Portuguese language) and back-translation (second translator) because these professionals were certified to perform translations, which therefore are officially attested for the entire territory of Brazil. This choice contributed towards improving the quality of the translated document, which is an important factor, because the document was thus officially recognized by public institutions and agencies and thus would be valid as an official or legal document.

Sworn translators are usually trained in the humanities and are approved for translating public edicts issued by the state boards of trade. Thus, such translators might be unacquainted with some medical terms, thus resulting in slight errors in translation. Such errors are expected, and therefore a review panel needs to be established in order to compare the original and translated versions before the process of content validation is started. In this manner, possible discrepancies can be resolved and the translation can be made fully understandable and can have satisfactory cross-cultural equivalence of scales.^8^ The assessment performed by the reviewers enabled adaptation of technical terms relating to drug classes and units of measurements, before the Delphi consensus technique was applied, without the risk of compromising the study.

In the first phase of the study, consensus was not attained for the cardiology-related statements describing the use of warfarin for chronic atrial fibrillation. The main studies conducted with new anticoagulant agents have compared these agents with warfarin and have aimed to assess the benefits and risks associated with these agents.^10^^,^^11^^,^^12^ However, most of these studies have been non-inferiority trials, i.e. comparative efficacy studies, which are performed to compare a new treatment with a traditional treatment in order to show that the new one is not inferior, but also not superior, to the existing treatment.^13^

Use of statins in cases with a known history of cerebral, peripheral or vascular disease, in which the patient remains functionally independent for activities of daily living and the life expectancy is more than five years, was also discussed and included for discussion in the second phase of the study. In this second phase, a consensus was achieved with regard to the evidence showing that the long-term benefit of statins in cerebral or peripheral atherosclerotic artery disease is poorer than that for coronary artery disease. Nevertheless, most of the participants agreed that statin therapy should be used, particularly when the low-density lipoprotein cholesterol (LDL-C) level is higher than 100 mg/dl.^12^^,^^14^^,^^15^^,^^16^^,^^17^

Furthermore, no consensus was reached in the first phase of the study in relation to the use of bisphosphonates in patients undergoing oral corticosteroid maintenance therapy. Bisphosphonates have antiresorptive action and increase bone mass. They are indicated for treatment and prevention of bone disorders. They are considered to be the first choice for treating osteoporosis and should be used together with calcium and vitamin supplements.^18^^,^^19^

A recent review failed to find any systematic reviews or meta-analyses of studies with three-year or longer follow-ups assessing fractures as outcomes associated with use of bisphosphonates. Nevertheless, case reports from Singapore and the United States have described occurrences of transverse fractures in the upper femoral shaft in patients treated with bisphosphonates. It is worth noting that most analyses on clinical trials or large datasets have failed to demonstrated higher total numbers of bone fractures among bisphosphonate users.^20^ In the second phase of the present study, the experts agreed on the use of bisphosphonates, following comments made by their peers. Just as in the case of warfarin, lack of critical analysis about the issue discussed might have contributed to the failure to achieve consensus in the first phase, because case reports do not suffice to exclude the use of medications with proven efficacy and safety in previously performed, well-designed clinical trials. Under such circumstances, case reports may be considered to be a possible source of information on adverse reactions to drugs, and they could be useful in guiding the monitoring of the agents involved.

Additionally, the score for regularly inhaled ß-2 agonists or anticholinergic agents in mild-to-moderate asthma or chronic obstructive pulmonary disease was less than the cutoff point of the 95% CI in the first phase of the study. It was therefore discussed again in the second phase. Some studies conducted among older adults have shown that ß-2 agonists might reduce the risk of exacerbation of asthma or chronic obstructive pulmonary disease, in addition to improving the patient’s survival.^19^ ß-2 agonists were also used among older adults with cardiovascular and chronic obstructive pulmonary disease, and the results indicated that these drugs did not seem to influence the occurrence of cardiac and pulmonary events or death in that population.^21^

The lower limit of the confidence interval regarding the use of fiber supplementation for chronic, symptomatic diverticular disease with constipation was less than the preset cutoff point, and so it was included in the second round of discussion. All of the participants agreed with this statement in the second phase. Use of fiber supplementation for treating diverticular disease has been adopted in several studies, in which fiber intake seemed to be associated with better outcomes in comparisons among individuals who ate fiber-rich foods. Use of fiber-rich foods has also been associated with the development of diverticular disease, given that the likelihood of developing this disease seemed to be higher among individuals eating low-fiber diets.^22^^,^^23^

The reproducibility of this study will be evaluated through the master’s degree project of one of the present authors.

### Limitations of the study

In cases in which only one participant disagreed with a given statement, but he or she did not provide appropriate justification, the discussions among the experts were rather shallow. This was because the participants were not provided with arguments against the use of a given medication and they merely held their previous positions due to the lack of new evidence requiring discussion. The Delphi consensus technique eliminates interpersonal factors that often influence consensus groups or committees, in which participants are face to face, and it encourages manifestation of honest opinions because of the lack of group pressure. The cost of applying the Delphi technique is low because there is no need for the participants to meet. The limitations of this technique derive from the doubts that are frequently cast on its scientific respectability, particularly regarding the selection and number of experts and the consensus criteria.^24^ To minimize these problems, a larger number of experts were invited to participate, in comparison with the original criteria.^7^ Regarding the consensus criteria, the cutoff point for accepting the experts’ opinions was established before the start of the study.

Another limitation is that the study was not designed to add new statements to START. Doing so could have added the ability to screen for possible prescription omissions that were not described in the original instrument.

## CONCLUSION

START was translated and adapted to Brazilian realities. Its validation by means of the Delphi consensus technique showed full agreement among the participants. START might be useful for other researchers and in clinical practice, with the aim of reducing the numbers of errors of omission with regard to prescriptions.
